# Engineering a Radiohybrid PSMA Ligand with an Albumin-Binding Moiety and Pharmacokinetic Modulation via an Albumin-Binding Competitor for Radiotheranostics

**DOI:** 10.3390/molecules30132804

**Published:** 2025-06-29

**Authors:** Saki Hirata, Hiroaki Echigo, Masayuki Munekane, Kenji Mishiro, Kohshin Washiyama, Takeshi Fuchigami, Hiroshi Wakabayashi, Kazuhiro Takahashi, Seigo Kinuya, Kazuma Ogawa

**Affiliations:** 1 Graduate School of Medical Sciences, Kanazawa University, Kakuma-machi, Kanazawa 920-1192, Ishikawa, Japan; s.hirata-059@stu.kanazawa-u.ac.jp (S.H.); h-echigo1010@stu.kanazawa-u.ac.jp (H.E.); munekane@p.kanazawa-u.ac.jp (M.M.); mishiro@p.kanazawa-u.ac.jp (K.M.); t-fuchi@p.kanazawa-u.ac.jp (T.F.); 2 Advanced Clinical Research Center, Fukushima Global Medical Science Center, Fukushima Medical University, 1 Hikarigaoka, Fukushima 960-1295, Fukushima, Japan; kwashi@fmu.ac.jp (K.W.); ktakahas@fmu.ac.jp (K.T.); 3 Department of Nuclear Medicine, Kanazawa University Hospital, Kanazawa University, Takara-machi 13-1, Kanazawa 920-8641, Ishikawa, Japan; wakabayashi@staff.kanazawa-u.ac.jp (H.W.); kinuya@med.kanazawa-u.ac.jp (S.K.)

**Keywords:** PSMA, albumin-binding moiety, astatine-211, radiotheranostics, targeted alpha therapy (TAT)

## Abstract

The prostate-specific membrane antigen (PSMA) is a well-established target for radiotheranostics in prostate cancer. We previously demonstrated that 4-(*p*-astatophenyl)butyric acid (APBA), an albumin-binding moiety (ABM) labeled with astatine-211 (^211^At), enables the modulation of pharmacokinetics and enhancement of therapeutic efficacy when combined with the post-administration of an albumin-binding competitor. However, this strategy has not been explored in PSMA-targeting ligands. We designed and synthesized [^211^At]**6**, a novel PSMA ligand structurally analogous to PSMA-617 with APBA. The compound was obtained via a tin–halogen exchange reaction from the corresponding tributylstannyl precursor. Comparative cellular uptake and biodistribution studies were conducted with [^211^At]**6**, its radioiodinated analog [^125^I]**5**, and [^67^Ga]Ga-PSMA-617. To assess pharmacokinetic modulation, sodium 4-(*p*-iodophenyl)butanoate (IPBA), an albumin-binding competitor, was administered 1 h postinjection of [^125^I]**5** and [^211^At]**6** at a 10-fold molar excess relative to blood albumin. The synthesis of [^211^At]**6** gave a radiochemical yield of 15.9 ± 7.7% and a radiochemical purity > 97%. The synthesized [^211^At]**6** exhibited time-dependent cellular uptake and internalization, with higher uptake levels than [^67^Ga]Ga-PSMA-617. Biodistribution studies of [^211^At]**6** in normal mice revealed a prolonged blood retention similar to those of [^125^I]**5**. Notably, post-administration of IPBA significantly reduced blood radioactivity and non-target tissue accumulation of [^125^I]**5** and [^211^At]**6**. We found that ABM-mediated pharmacokinetic control was applicable to PSMA-targeted radiotherapeutics, broadening its potential for the optimization of radiotheranostics.

## 1. Introduction

Prostate cancer is the second most frequent cancer disease in men worldwide and the fifth leading cause of male cancer-related death in 2022 [1]. The 5-year survival rate for patients with non-metastatic prostate cancer approaches approximately 100%; however, in cases of patients with distant metastases, the survival rate decreases to 37% [2]. In recent years, prostate-specific membrane antigen (PSMA)-targeted radiopharmaceuticals have gained significant attention [3]. As a type-II transmembrane glycoprotein, the PSMA is highly expressed on prostate cancer cells. The increase in the expression level of the PSMA correlates with the malignancy of prostate cancer, so it is an essential target for molecular targeted imaging and therapy [4,5,6]. Several diagnostic and therapeutic agents based on the Lys-urea-Glu pharmacophore have been developed to have high affinity for the PSMA [7,8,9,10]. In 2020, [^68^Ga]Ga-PSMA-11 was approved by the US Food and Drug Administration (FDA) as the first radiopharmaceutical for the positron emission tomography (PET) imaging of PSMA-positive lesions. Moreover, the therapeutic radioligand [^177^Lu]Lu-PSMA-617 received approval in 2022 because it was associated with a higher prostate-specific antigen (PSA) response and fewer adverse events than those of the standard of care for patients with metastatic castration-resistant prostate cancer (mCRPC) [11,12]. In nuclear medicine, [^68^Ga]Ga-PSMA-11 is commonly used for molecular imaging prior to targeted endoradionuclide therapy with [^177^Lu]Lu-PSMA-617. The primary purpose of PET or single-photon emission computed tomography (SPECT) diagnostic imaging is to detect tumor lesions and to assess the potential benefit of the planned therapy [13,14]. For an accurate prediction of therapeutic outcomes, it is ideal that the diagnostic and therapeutic radiopharmaceuticals exhibit similar pharmacokinetic profiles before therapy [15].

**Figure 1:**
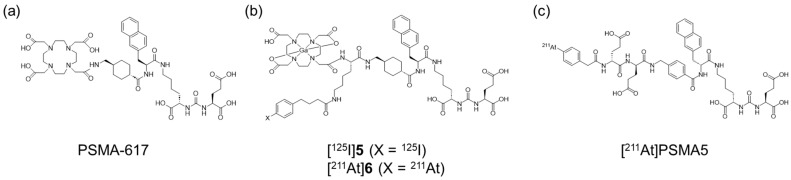
Chemical structures of (**a**) PSMA-617, (**b**) [^125^I]**5** and [^211^At]**6,** and (**c**) [^211^At]PSMA5.

Recently, PSMA-targeted alpha therapy (TAT) has been reported as a more beneficial method for mCRPC patients with poor response to [^177^Lu]Lu-PSMA-617 [16]. Radiopharmaceuticals for TAT targeting the PSMA, such as [^225^Ac]Ac-PSMA-617 (Figure 1a) and [^211^At]PSMA5 (Figure 1c), have demonstrated the desired therapeutic effects in clinical or preclinical studies [17,18]. However, their rapid blood clearance could limit the tumor retention and therapeutic efficacy of PSMA ligands. To enhance therapeutic efficacy and reduce adverse effects, several approaches are currently being investigated for Lys-urea-Glu-based PSMA ligands. The introduction of an albumin-binding moiety (ABM) into PSMA ligands has been shown to enhance integral tumor uptake by prolonging blood circulation time [19,20]. However, this may also lead to undesired retention in normal tissues depending on the albumin-binding affinity [20]. One well-known potent ABM with competitive displacement ability from albumin is 4-(*p*-iodophenyl)butyric acid (IPBA) [21], and various PSMA ligands with IPBA have been shown to exhibit higher accumulation and longer retention in tumor tissues than those in PSMA ligands without IPBA [22,23,24,25,26]. Among these, PSMA-ALB-53, developed by the Müller group, is a representative albumin-binding PSMA ligand that incorporates IPBA as the ABM [27]. In this study, we used the same compound (PSMA-ALB-53) as compound 7, serving as a reference lead compound for the design of radiohalogenated and radiometal-labeled derivatives to evaluate pharmacokinetic modulation strategies. Additionally, ^211^At, which is an alpha-emitting radionuclide, has recently received significant attention due to its favorable physical and chemical properties. However, no ^211^At-labeled PSMA ligand for TAT with improved pharmacokinetics through incorporating ABM has been reported to date.

In our previous study, we evaluated 4-(*p*-astatophenyl)butyric acid (APBA), synthesized by substituting the iodine in IPBA with ^211^At by the introduction of [^211^At]APBA into the Arg-Gly-Asp (RGD) peptide [28]. The ^211^At-labeled RGD peptide with APBA increased tumor accumulation and showed significant tumor growth inhibition compared with a vehicle control group, indicating that APBA functioned as ABM, similar to IPBA. However, the clearance of the APBA-introduced RGD peptide from blood and normal tissues was too slow and unfavorable for efficient treatment with ^211^At because its physical half-life (7.2 h) is comparatively short for a therapeutic radionuclide. Therefore, we attempted to optimize the pharmacokinetics of the APBA-introduced RGD peptide by post-administering a compound with a high affinity for albumin (an albumin-binding competitor) and successfully achieved competitive displacement from albumin [29].

This study aimed to determine if APBA introduced into a PSMA ligand can also function as an ABM and if the strategy to optimize pharmacokinetics by post-administration of an albumin-binding competitor could be effective. We designed, synthesized, and evaluated the radiohybrid compounds **5** and **6** as PSMA ligands (Figure 1b). These compounds possess both radiohalogen and radiometal labeling sites and are structurally similar to PSMA-617 as a PSMA ligand. Furthermore, **5** and **6** have IPBA and APBA, respectively, providing both a functional molecule for ABM and a radiohalogen labeling site (Figure 1). Although we aimed to develop ^68^Ga (T_1/2_: 67.8 min)-labeled agents for PET and ^123^I (T_1/2_: 13.2 h)-labeled agents for SPECT, we used ^67^Ga (T_1/2_: 3.3 d) and ^125^I (T_1/2_: 59.4 d). This substitution was due to the limited availability of ^68^Ga and ^123^I at our facility, as well as the advantages offered by the longer half-lives of ^67^Ga and ^125^I, which enabled more flexible and prolonged preclinical evaluations. Although these radionuclides are not intended for clinical imaging applications, they serve as suitable surrogates for the initial characterization of the radiohybrid compounds.

## 2. Results and Discussion

### 2.1. Synthesis and Radiolabeling

**Scheme 1:**
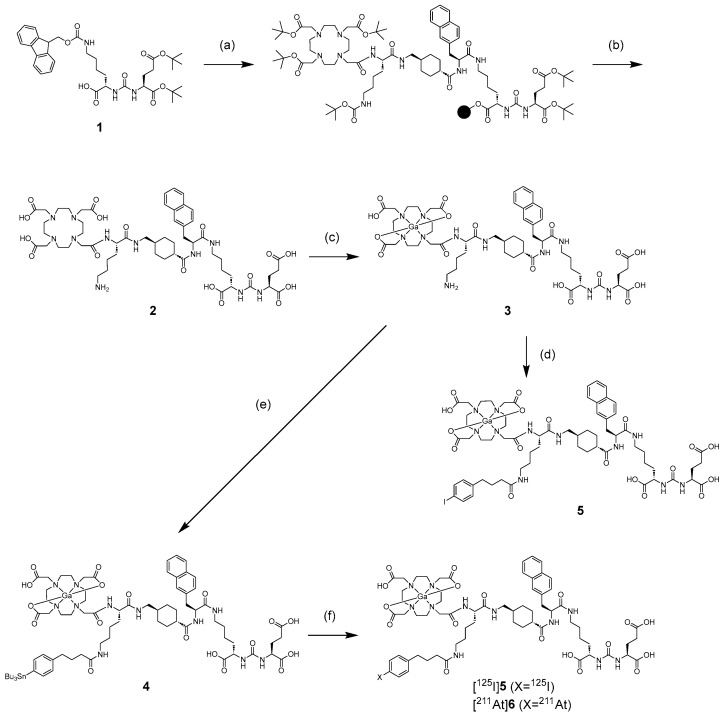
Synthetic scheme of **4**, **5**, [^125^I]**5**, and [^211^At]**6**. Reagents: (**a**) (i) 2-chlorotrityl chloride resin, Glu(O*t*Bu)_2_-urea-Lys(Fmoc)-OH, DIPEA, DCM, 2 h, room temperature (rt); (ii) 20% piperidine/DMF, 15 min, rt; (iii) Fmoc-amino acid or Tris-DOTA, HOBt, *N,N*-diisopropylcarbodiimide (DIC), DMF, 2 h, rt. (**b**) trifluoroacetic acid (TFA), TIS, H_2_O (95/2.5/2.5 *v*/*v*/*v*), 2 h, rt. (**c**) Ga(NO_3_)_3_, 3 M ammonium acetate buffer (pH 5), 1 h, 40 °C. (**d**) 4-(*p*-iodophenyl)butanoate NHS ester, DIPEA, DMF, 5 h, rt. (**e**) 4-(*p*-tributylstannylphenyl)butanoate NHS ester, DIPEA, DMF, 4 h, rt. (**f**) [^125^I]NaI/[^211^At]At^−^, chloramine-T, AcOH, MeOH, 15 min, 60 °C.

**Figure 2:**
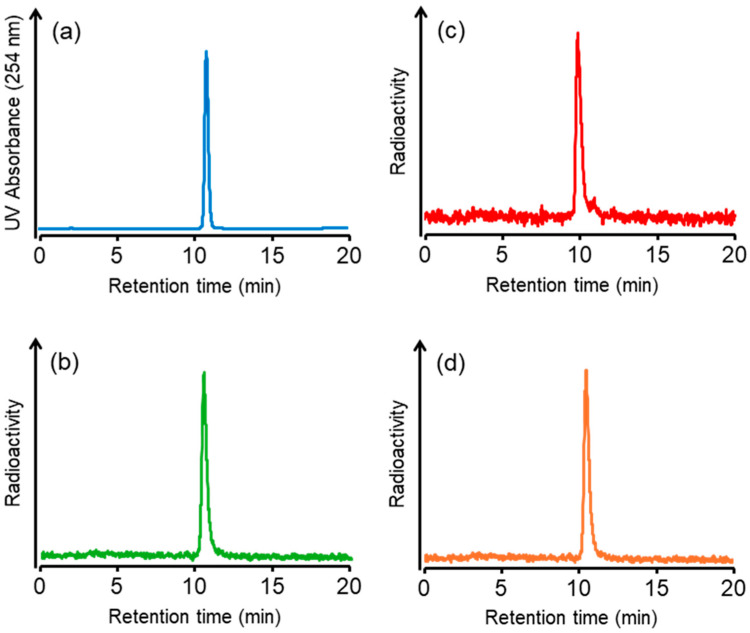
HPLC chromatograms of (**a**) **5**, (**b**) [^125^I]**5**, (**c**) [^211^At]**6**, and (**d**) [^67^Ga]**5**. HPLC condition: a flow rate of 1 mL/min with a gradient mobile phase of 60% methanol in water with 0.1% TFA to 80% methanol in water with 0.1% TFA for 20 min.

We synthesized a tributylstannyl precursor **4** for [^125^I]**5** and [^211^At]**6** according to the procedure outlined in Scheme 1. Compound **4** was obtained from the Fmoc solid-phase synthesis. After deprotection of the protected groups under acidic conditions, Ga complexation was performed. Conjugation with the tributylstannylated albumin-binding moiety to the amino group in **3** yielded **4** with a total yield of 8%. The radioiodination of **4** by the tin–halogen exchange reaction using *N*-chlorosuccinimide (NCS) as an oxidant was performed according to the method described in our previous report [28]. However, the radiolabeling reaction did not proceed, so the oxidant was changed from NCS to chloramine-T, which gave [^125^I]**5** at a 31.7 ± 12.2% (*n* = 8) radiochemical yield. In the case of ^211^At-labeling, [^211^At]**6** was obtained in 15.9 ± 7.7% (*n* = 4) radiochemical yield under the same conditions as for radioiodination. The identity of [^125^I]**5** was verified by comparing the retention time with that of the corresponding nonradioactive iodinated compound **5** (Figure 2). We also identified [^211^At]**6** by comparing the retention time with that of **5** because there are no stable isotopes for astatine. The radiochemical purities of [^125^I]**5** and [^211^At]**6** were > 97% after RP-HPLC purification. Moreover, we also confirmed that **7** could be radiolabeled with a radiometal, ^67^Ga. The radiochemical yield of [^67^Ga]**5** was 70%, and the radiochemical purity of [^67^Ga]**5** was > 96% after RP-HPLC purification. As HPLC purification completely separated the radiolabeled compounds from the precursors, the molar activities for [^125^I]**5**, [^211^At]**6**, and [^67^Ga]**5** were 81.3 GBq/µmol, 16.1 TBq/µmol, and 1.5 TBq/µmol, respectively. The radiolabeling method of [^67^Ga]**5** is shown in the Supplementary Information. All radioligands were stable in 0.1 M phosphate buffer at 37 °C for at least 24 h, as confirmed by HPLC analysis (data provided in the Supplementary Information). These results indicate that the labeling strategies employed are suitable for subsequent in vitro and in vivo evaluations.

### 2.2. Cellular Uptake Experiments

**Figure 3:**
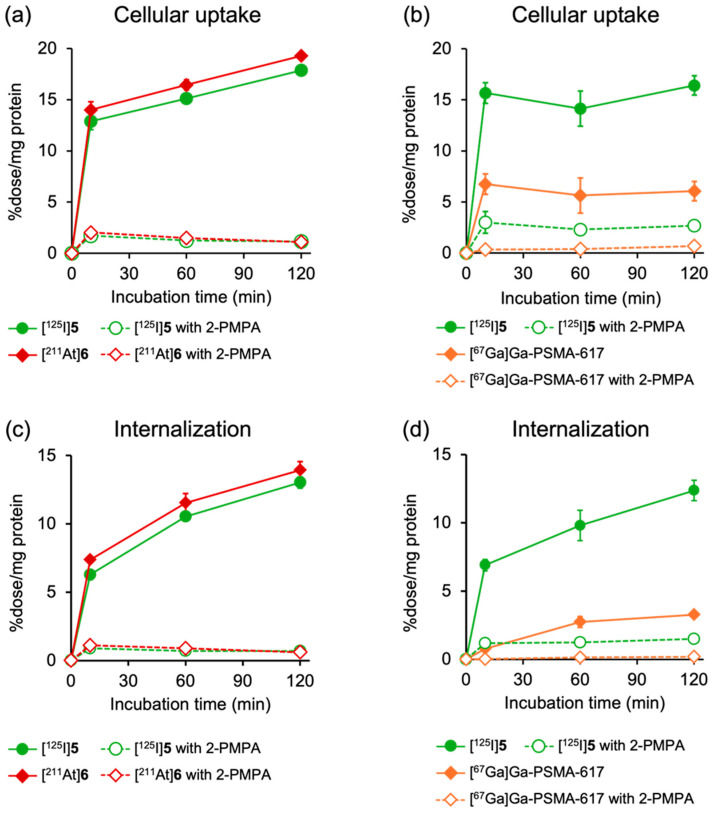
Time-dependent cellular uptake of (**a**) [^125^I]**5** and [^211^At]**6** and (**b**) [^125^I]**5** and [^67^Ga]Ga-PSMA-617, as well as internalization of (**c**) [^125^I]**5** and [^211^At]**6** and (**d**) [^125^I]**5** and [^67^Ga]Ga-PSMA-617 in LNCaP cells. Specific binding of PSMA is blocked by excess amount (100 µM) of 2-PMPA. Data are presented as mean ± SD for three samples.

Figure 3 shows the results of cellular uptake experiments using PSMA-expressing LNCaP cells. In this study, [^125^I]**5** and [^211^At]**6**, [^67^Ga]Ga-PSMA-617 and [^125^I]**5**, or [^125^I]**5** and [^67^Ga]**5** were co-incubated into the cells to minimize experimental errors. [^125^I]**5** and [^211^At]**6** and [^67^Ga]**5** and [^125^I]**5** exhibited similar uptake and internalization profiles at all timepoints, and their uptakes were significantly reduced by the addition of excess 2-PMPA (100 µM), a potent and selective inhibitor of the PSMA (Figure 3a,c and Figure S1). All tracers demonstrated rapid uptakes and a time-dependent increase in internalization into LNCaP cells, whereas the cellular uptake and internalization levels were lower for [^67^Ga]Ga-PSMA-617 than for [^125^I]**5** and [^211^At]**6**. Modification with an ABM between the DOTA chelator and the PSMA-binding site of PSMA-617 reportedly had minimal effects on the binding affinity for the PSMA [30]. Furthermore, the uptake of [^125^I]**5** and [^67^Ga]Ga-PSMA-617 was similarly inhibited by 2-PMPA, with an inhibition rate of 83.6% and 88.6%, respectively. The finding suggests that the higher cellular uptakes of [^125^I]**5** and [^211^At]**6** than that of [^67^Ga]Ga-PSMA-617 could be attributed to the enhanced PSMA-mediated uptake efficiency by the presence of ABM. Consistent with these results, previous studies on PSMA derivatives incorporating ABM, such as IPBA or Evans Blue, have demonstrated higher uptake by PSMA-expressing cells than by PSMA-617, despite exhibiting similar binding affinities for the PSMA [31,32,33].

### 2.3. Biodistribution in Normal Mice

In this study, [^67^Ga]Ga-PSMA-617 and [^125^I]**5** or [^125^I]**5** and [^211^At]**6** were intravenously co-injected into tumor-bearing mice. The double-trace experiments can minimize the number of mice and experimental errors.

**Figure 4:**
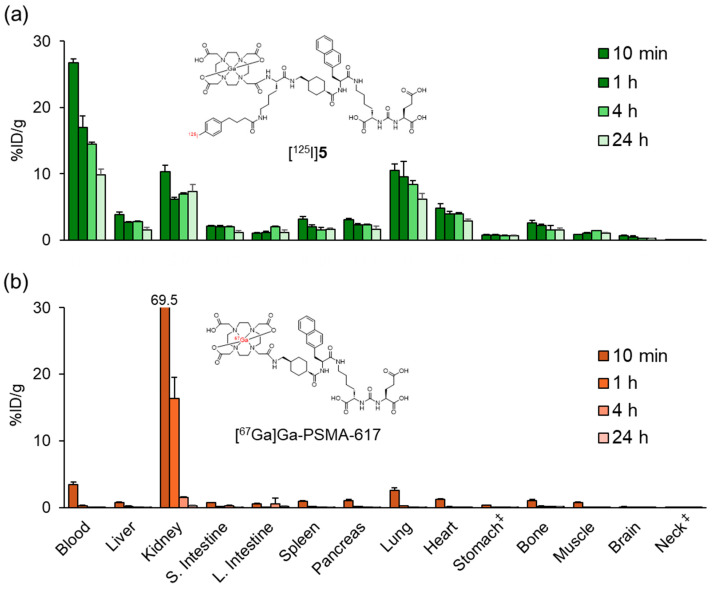
Biodistribution of radioactivity after the intravenous injection of (**a**) [^125^I]**5** and (**b**) [^67^Ga]Ga-PSMA-617 in normal mice. S. Intestine and L. Intestine indicate the small intestine and large intestine, respectively. ^‡^ Expressed as %ID. Each value represents the mean (SD) for three animals. The distribution data of [^125^I]**5** in Figure 4 and Figure 5 were evaluated for each experiment.

Figure 4 and Table S1 show the biodistribution results of [^67^Ga]Ga-PSMA-617 and [^125^I]**5** in normal mice. While [^67^Ga]Ga-PSMA-617 exhibited rapid clearance from most organs except the kidney at 1 h postinjection, [^125^I]**5** showed significantly prolonged blood retention, resulting in high accumulation in the lung and heart. At 24 h postinjection, [^67^Ga]Ga-PSMA-617 was primarily excreted in the urine with minimal fecal excretion (urine: 58.0 ± 2.6%ID, feces: 2.4 ± 0.3%ID), whereas [^125^I]**4** demonstrated significantly lower total excretion (urine: 10.3 ± 0.7%ID, feces: 2.3 ± 0.3%ID). These results showed a trend similar to the changes in pharmacokinetics observed when ABM was introduced into the RGD peptide in our previous study [28].

**Figure 5:**
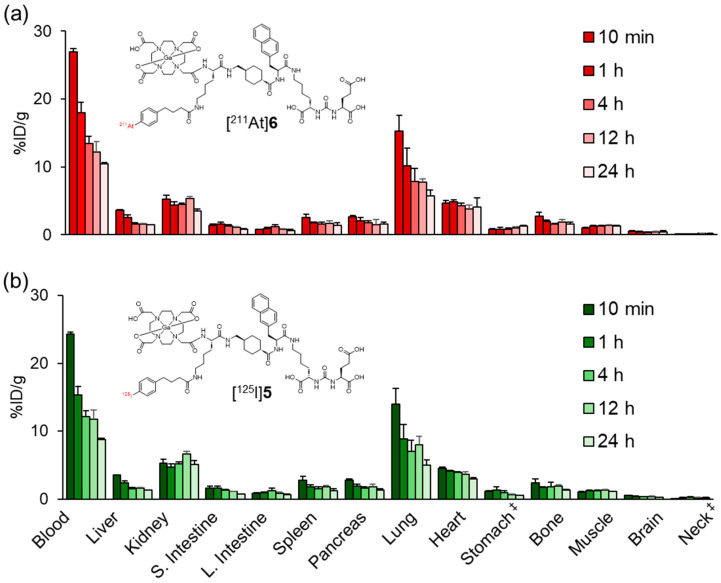
Biodistribution of radioactivity after the intravenous injection of (**a**) [^211^At]**6** and (**b**) [^125^I]**5** in normal mice. S. Intestine and L. Intestine indicate the small intestine and large intestine, respectively. ^‡^ Expressed as %ID. Each value represents the mean (SD) for three or four animals. The distribution data of [^125^I]**5** in Figure 4 and Figure 5 were evaluated for each experiment.

The biodistribution profile of [^211^At]**6** resembled that of [^125^I]**5** with prolonged blood retention, suggesting that the APBA introduced into PSMA ligands also functions as an ABM (Figure 5 and Table S2). Meanwhile, low levels of radioactivity were observed in the stomach and neck region, which includes the thyroid, indicating that minimal deiodination or deastatination occurred for both [^125^I]**5** and [^211^At]**6**. These findings further support the in vivo stability of the halogenated compounds. The radioactivity levels in the blood, lung, and heart of [^211^At]**6** were slightly higher than those of [^125^I]**5**. In our previous study, blood retention was higher for the APBA-introduced RGD peptide than for the IPBA-introduced RGD peptide [28,29]. The albumin-binding affinity of ABMs containing halogen groups on the benzene ring of the phenylpropyl moiety has been reported to correlate with their lipophilicity, following the order I > Br > Cl > F based on in vivo biodistribution trends [34]. However, Kuo et al. did not directly measure albumin-binding affinity. More recently, Brandt et al. provided experimentally determined dissociation constants and structure–affinity relationships, confirming this general trend [35]. Based on the results from our current and previous studies, we propose that this trend can be extended to include astatine, following the order At > I > Br > Cl > F.

**Figure 6:**
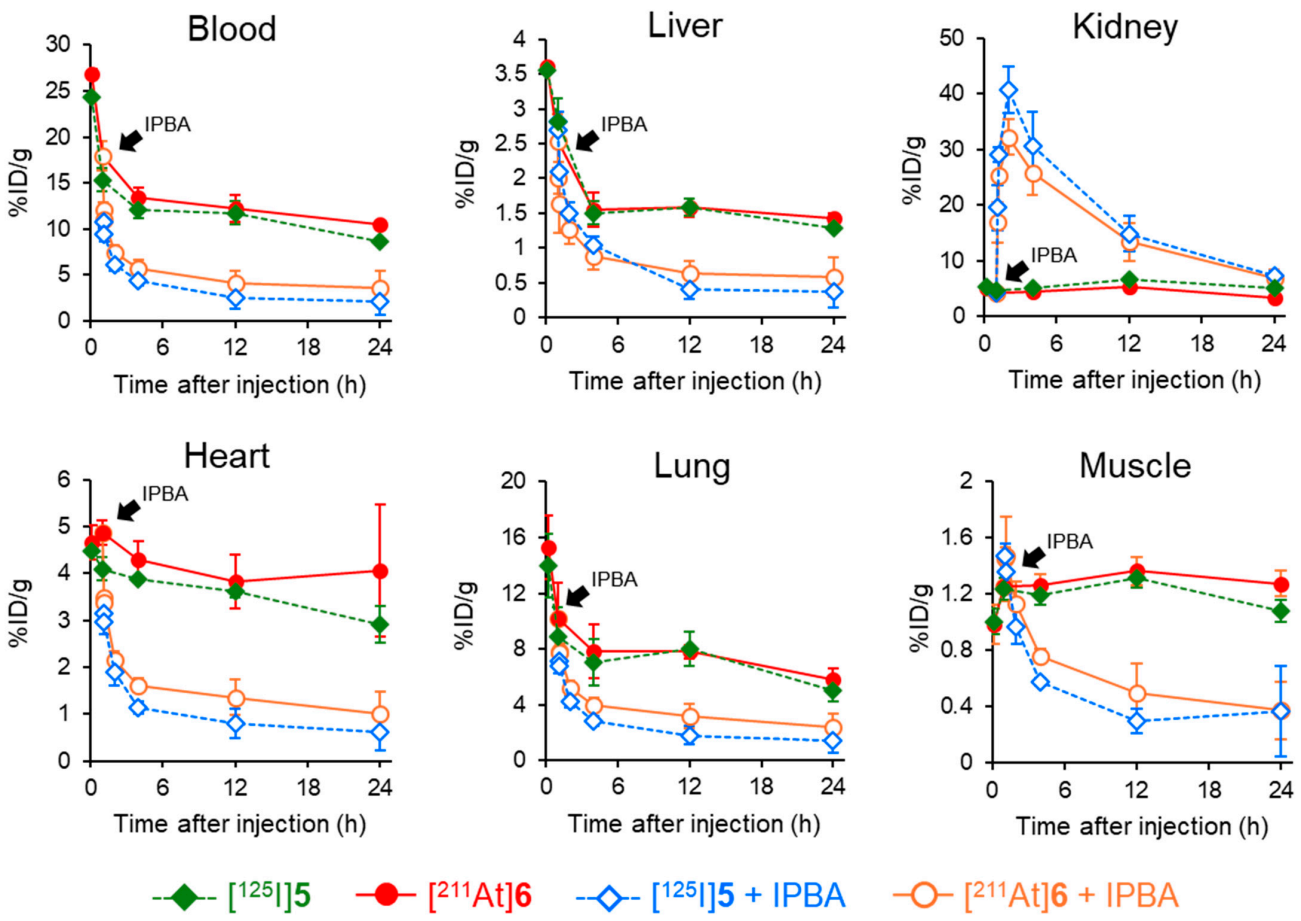
Comparison of the biodistribution in normal mice with or without administering IPBA at 1 h postinjection of [^125^I]**5** and [^211^At]**6**.

The PSMA ligand designed in this study contains not only an ABM as a radiohalogen labeling site but also a DOTA chelator, enabling stable coordination with various radiometals. The structural design permits the use of various therapeutic radionuclides, such as Lu-177 and Ac-225. Although the delayed blood clearance resulting from the ABM-based drug design is advantageous for radionuclides with relatively long half-lives, such as Lu-177 (T_1/2_: 6.64 d) and Ac-225 (T_1/2_: 9.92 d), it is unsuitable for radionuclides with short half-lives, such as At-211 (T_1/2_: 7.21 h) or Ga-68 (T_1/2_: 67.7 min), which require rapid diagnostic imaging or efficient therapy after administration. In our previous study using the RGD peptide, we addressed this issue by demonstrating that the additional administration of IPBA, an albumin-binding competitor, promoted blood clearance after injecting radioligands containing an ABM. This effect is attributed to the competitive inhibition of the interaction between the radioligands and albumin. By administering an albumin-binding competitor, blood clearance was promoted in a dose-dependent manner. Consistent with the reduction in blood radioactivity, significant decreases in radioactivity were also observed in other non-targeted tissues, whereas tumor accumulation was maintained in tumor-bearing mice [29]. Based on these findings, biodistribution experiments of [^125^I]**5** and [^211^At]6 with the administration of an albumin-binding competitor were conducted in normal mice. (Figure 6 and Table S3). The radioactivity levels of [^125^I]**5** and [^211^At]**6** were significantly decreased by IPBA administration except in the kidney. This higher kidney radioactivity was thought to result not only from enhanced renal excretion due to IPBA administration but also from the PSMA expressed in the kidney [36].

Several albumin-binding PSMA ligands, including PSMA-ALB-53 and PSMA-ALB-56, have been reported to enhance tumor uptake and therapeutic efficacy by prolonging systemic circulation, particularly when labeled with therapeutic radionuclides such as ^177^Lu or ^225^Ac [27,37]. However, this prolonged circulation is often accompanied by an increased absorbed dose in off-target organs such as the kidneys and bone marrow. These earlier studies utilized static ABM structures that lacked the capacity for pharmacokinetic modulation after administration and were not optimized for use with short-lived radionuclides. To address these limitations, the present study introduces, for the first time, a pharmacokinetic modulation strategy for an ^211^At-labeled PSMA-targeting ligand using IPBA, an albumin-binding competitor. This approach adds a dynamic element to ABM-based design, enabling time-controlled clearance and potentially reducing off-target exposure while maintaining tumor accumulation. By allowing temporal separation between systemic circulation and clearance, this strategy offers tunable pharmacokinetics, which may be particularly advantageous for short-lived theranostic agents. This represents a promising direction for improving the balance between tumor uptake and background reduction in PSMA-targeted radiotherapy.

One of the limitations of this study was that, due to the challenges in establishing a stable tumor-bearing mouse model and the limited availability of At-211, only normal mice were used for evaluation, and a direct assessment of tumor accumulation was not possible. In our previous study, the administration of IPBA as an albumin-binding competitor temporarily enhanced tumor accumulation, despite concerns that it might reduce the tumor uptake of the ^211^At-labeled RGD peptide. Both RGD and PSMA ligands are internalized into cells after binding to their respective targets on the plasma membrane. This similarity in internalization mechanisms suggests that the transient enhancement of tumor accumulation by IPBA observed with RGD peptides might also apply to PSMA-targeting ligands. Future studies using tumor-bearing models will be essential to validate this hypothesis and to further evaluate the tumor uptake, pharmacokinetics, and therapeutic efficacy of [^211^At]**6**, as well as the imaging performance of [^125^I]**5** or [^67/68^Ga]**5** as surrogate compounds. The optimization of the timing schedule for IPBA administration will also be an important focus for future research. Fine-tuning this parameter may help maximize tumor uptake while minimizing off-target accumulation, thereby enabling a more selectively absorbed dose to the tumor. This approach could ultimately enhance the therapeutic efficacy of ^211^At-labeled compounds incorporating ABM while minimizing damage to healthy tissues.

## 3. Materials and Methods

### 3.1. General

All chemical reagents and solvents were purchased commercially from Tokyo Chemical Industry, Co., Ltd. (Tokyo, Japan), Merck (Darmstadt, Germany), Nacalai Tesque, Inc. (Kyoto, Japan), Fujifilm Wako Pure Chemical Corporation (Osaka, Japan), Kokusan Chemical Co., Ltd. (Tokyo, Japan), Ambeed, Inc (Arlington Hts, IL, USA), and Watanabe Chemical Industries, Ltd. (Hiroshima, Japan). [^125^I]Sodium iodide (629 GBq/mg) was purchased from Revvity (Waltham, MA, USA). [^211^At]At^−^ was produced on a CYPRIS MP-30 cyclotron (Sumitomo Heavy Industries, Ltd., Tokyo, Japan) in the Advanced Clinical Research Center at Fukushima Medical University via a ^209^Bi(α, 2n)^211^At nuclear reaction and transported to Kanazawa University [38,39]. [^67^Ga]GaCl_3_ was prepared according to a previous report with modifications [40].

A [^67^Ga]Ga-citrate solution (Nihon Medi-Physics Co., Ltd., Tokyo, Japan) (74 MBq, 1 mL, pH 6.0–8.0) was passed through a Sep-Pak^®^ Silica Plus Light Cartridge (Waters, Co., Ltd., Milford, MA, USA) preconditioned with 1 mL of ultrapure water. The cartridge was washed twice with 1 mL of ultrapure water to remove citrate ions. The retained radioactivity on the cartridge was eluted with 1 mL of 0.1 M HCl, yielding a [^67^Ga]GaCl_3_ solution free of citrate ions, suitable for subsequent radiolabeling applications. The recovery rate of [^67^Ga]GaCl_3_ was 77.6 ± 7.8% (*n* = 6), expressed as the percentage of eluted ^67^Ga radioactivity relative to the total radioactivity of the [^67^Ga]Ga-citrate solution passed through the Sep-Pak^®^ Silica Plus Light Cartridge. The radiochemical purity of the [^67^Ga]GaCl_3_ solution was determined using instant radio thin-layer chromatography on silica gel (ITLC-SG) (Agilent Technologies, Santa Clara, CA, USA), using 0.4 M sodium acetate buffer at pH 4 as a mobile phase. Rf values for [^67^Ga]GaCl_3_ and [^67^Ga]Ga-citrate were 0 and 1, respectively [40]. The radiochemical purity was greater than 99% in all cases.

[^67^Ga]Ga-PSMA-617 was synthesized according to a previous report [30]. Mass spectrometry was performed on LCMS-9030 (Shimazu Corp, Kyoto, Japan). An analytical high-performance liquid chromatography (HPLC) system was obtained on the Shimadzu LC-20AD pump, SPD-20A UV detector at a wavelength of 220 nm, and CTO-20A column oven maintained at 40 °C (Shimadzu Corp., Kyoto, Japan). LNCaP prostate cancer cells were obtained from the Institute of Development, Ageing and Cancer, Tohoku University (Sendai, Japan). Other reagents were of reagent grade and used as received.

### 3.2. Synthesis

Compounds **4**, **5**, [^125^I]**5**, and [^211^At]**6** were synthesized according to the procedure outlined in Scheme 1.

#### 3.2.1. Glu(OtBu)_2_-Urea-Lys(Fmoc)-OH (**1**)

Glu(O*t*Bu)_2_-urea-Lys(Fmoc)-OH (**1**) was synthesized according to our previous report [30].

#### 3.2.2. DOTA-K-TXA-Nal-KuE (**2**)

1,4,7,10-Tetraazacyclododecane-1,4,7,10-tetraacetic acid (DOTA)-K-TXA-Nal-KuE (**2**) was synthesized by conventional fluorenylmethoxycarbonyl (Fmoc)-based solid phase methodology according to a previous report with slight modifications [30]. After cleaving the peptide from the resin and deprotecting the protecting groups for a 2 h reaction at room temperature by a mixture of 95% TFA, 2.5% water, and 2.5% triisopropylsilane (TIS), the mixture was filtered and concentrated by nitrogen gassing. The residue was purified by reversed-phase high-performance liquid chromatography (RP-HPLC) using a Cosmosil 5C_18_-AR-II (10 × 150 mm) at a flow rate of 4 mL/min with a gradient mobile phase of 20% methanol in water with 0.1% TFA to 60% methanol in water with 0.1% TFA for 20 min with UV detection at 254 nm. (Retention time: 7.2 min.) The solvent was removed by lyophilization to yield DOTA-K-TXA-Nal-KuE (**2**) (37.8 mg, 24%) as a colorless solid.

DOTA-K-TXA-Nal-KuE (**2**): HRMS (ESI^+^) (*m/z* calcd. for C_55_H_85_N_11_O_17_ [M+2H]^2+^): 585.8057 found: 585.8060.

#### 3.2.3. Ga-DOTA-K-TXA-Nal-KuE (**3**)

DOTA-K-TXA-Nal-KuE (**2**) (3.0 mg, 2.6 µmol, 1 equiv) was dissolved in 1.0 mL of 3 M ammonium acetate buffer (pH 5.0), and Ga(NO_3_)_3_ (6.5 mg, 25.6 µmol, 10 equiv) was added to the solution. The mixture was stirred at 40 °C for 1 h and purified by RP-HPLC performed with a Cosmosil 5C_18_-AR-II (10 × 150 mm) at a flow rate of 4 mL/min with a gradient mobile phase of 40% methanol in water with 0.1% TFA to 50% methanol in water with 0.1% TFA for 10 min with UV detection at 254 nm. (Retention time: 6.1 min.) The solvent was removed by lyophilization to yield Ga-DOTA-K-TXA-Nal-KuE (**3**) (1.3 mg, 38%) as a colorless solid.

Ga-DOTA-K-TXA-Nal-KuE (**3**): HRMS (ESI^+^) (*m/z* calcd. for C_55_H_82_GaN_11_O_17_ [M+H]^+^): 1237.5140 found: 1237.5101.

#### 3.2.4. 4-(*p*-Tributylstannylphenyl)butanoate NHS Ester and 4-(*p*-Iodophenyl)butanoate NHS Ester

4-(*p*-Tributylstannylphenyl)butanoate NHS ester and 4-(*p*-iodophenyl)butanoate NHS ester were synthesized according to our previous report [28].

#### 3.2.5. Ga-DOTA-K[4-(*p*-Tributylstannylphenyl)butyryl]-TXA-Nal-KuE (**4**)

Ga-DOTA-K-TXA-Nal-KuE (**3**) (1 mg, 0.8 µmol, 1 equiv) was dissolved in 100 µL of DMF, and then 4-(*p*-tributylstannylphenyl)butanoate NHS ester (4.4 mg, 8 µmol, 10 equiv) and DIPEA (2.7 µL, 16 µmol, 20 equiv) were added to the solution. The mixture was stirred at room temperature for 4 h and purified by RP-HPLC performed with Cosmosil 5C_18_-AR-II (10 × 150 mm) at a flow rate of 4 mL/min with a gradient mobile phase of 80% methanol in water with 0.1% TFA to methanol with 0.1% TFA for 10 min with UV detection at 254 nm. (Retention time: 8.4 min.) The solvent was removed by lyophilization to yield Ga-DOTA-K[4-(*p*-tributylstannylphenyl)butryl]-TXA-Nal-KuE (**4**) (0.8 mg, 60%) as a colorless oil.

DOTA-K[4-(*p*-tributylstannylphenyl)butyryl]-TXA-Nal-KuE (**4**): HRMS (ESI^−^) (*m/z* calcd. for C_77_H_115_N_11_O_18_Sn [M−2H]^2−^): 835.3355 found: 835.3342.

#### 3.2.6. Ga-DOTA-K(IPBA)-TXA-Nal-KuE (**5**)

Ga-DOTA-K-TXA-Nal-KuE (**3**) (1.1 mg, 0.89 µmol, 1 equiv) was dissolved in 100 µL of DMF, and then 4-(iodophenyl)butanoate NHS ester (3.4 mg, 8.9 µmol, 10 equiv) and DIPEA (3.0 µL, 17.8 µmol, 20 equiv) were added to the solution. The mixture was stirred at room temperature for 5 h and purified by RP-HPLC performed with Cosmosil 5C_18_-AR-II (10 × 150 mm) at a flow rate of 4 mL/min with a gradient mobile phase of 60% methanol in water with 0.1% TFA to 80% methanol in water with 0.1% TFA for 20 min with UV detection at 254 nm. (Retention time: 10.4 min.) The solvent was removed by lyophilization to yield Ga-DOTA-K(IPBA)-TXA-Nal-KuE (**5**) (1.2 mg, 90%) as a colorless solid.

Ga-DOTA-K(IPBA)-TXA-Nal-KuE (**3**): HRMS (ESI^−^) (*m/z* calcd. for C_65_H_88_GaIN_11_O_18_ [M−2H]^2−^): 753.2310 found: 753.2317.

### 3.3. Radiolabeling

The preparation of [^125^I]**5** and [^211^At]**6** was performed by electrophilic halogenation using the corresponding tributylstannyl precursor; **4**. 25 µg of compound **4** was dissolved in 10 µL of methanol in the reaction vial. The mixture of 3 µL of acetic acid, 25 µL of chloramine-T in methanol (10 mg/mL), and 10 µL of [^125^I]NaI (3.7 MBq) or [^211^At]At^−^ (3.7 MBq) solution were added to the vial. After heating at 60 °C for 15 min ([^125^I]**5**), the mixture was quenched by 30 µL of aqueous NaHSO_3_ (10 mg/mL) and purified by RP-HPLC using Cosmosil 5C_18_-AR-II (4.6 × 150 mm) at a flow rate of 1 mL/min with a gradient mobile phase of 60% methanol in water with 0.1% TFA to 80% methanol in water with 0.1% TFA for 20 min. After RP-HPLC purification, the radiolabeled compounds ([^125^I]**5**, [^211^At]**6**, and [^67^Ga]**5**) were collected in a water/methanol solution containing 0.1% TFA. To remove residual methanol and TFA, the solvent was evaporated under a gentle stream of nitrogen gas until only a minimal volume of water remained. The radioligands were then reconstituted in normal saline or cell culture medium as appropriate for in vitro and in vivo studies. The pH of the final formulation was measured and confirmed to be within the physiological range, indicating that TFA was effectively removed. The volatility of TFA and methanol under these conditions suggests that their residual levels were negligible. This procedure ensured that the radioligands were in a suitable formulation for subsequent biological evaluation. The molar activities were estimated based on the theoretical maximum values. [^125^I]NaI, [^211^At]At^−^, and [^67^Ga]GaCl_3_ were obtained under no-carrier-added conditions, and unreacted precursors were completely removed by HPLC, justifying the use of theoretical molar activity values.

### 3.4. Cellular Uptake Experiments

Cellular uptake experiments were performed according to a previous report with slight modifications [24,28]. LNCaP cells expressing PSMA were cultured in RPMI 1640 medium containing 10% fetal bovine serum (FBS) and 1% penicillin–streptomycin on 6-well culture plates (containing 5 × 10^5^ cells/well) for 24 h in a humidified atmosphere (5% CO_2_) incubator at 37 °C. After removing the medium and washing with phosphate-buffered saline (PBS) (−) once, a mixed solution of [^125^I]**5** and [^211^At]**6**, [^125^I]**5** and [^67^Ga]Ga-PSMA-617, and [^125^I]**5** and [^67^Ga]**5** in 1.0 mL of medium without FBS was added (3.7 kBq/each tracer/well). The final concentrations of the radioligands for [^125^I]**5**, [^211^At]**6**, and [^67^Ga]**5** were 45.5 pM, 0.2 pM, and 2.5 pM, respectively. These solutions containing radioligands were diluted in saline containing 0.05% (w/v) bovine serum albumin (BSA) to prevent adsorption to tubes or plates and further diluted with medium to 0.00125% as the final BSA concentration. After incubation for 10, 60, and 120 min, the medium from each well was removed, and the cells were washed twice with ice-cold PBS (−); the surface of the cells was washed twice with ice-cold 0.2 M glycine buffer (pH 3.0). The cells were lysed using a 1 M NaOH aqueous solution. The radioactivity of ^67^Ga,^125^I and ^211^At in the 1 M NaOH aqueous solution was determined and defined as the internalized radioligand fraction using an auto well gamma counter (ARC-8001, Hitachi, Ltd., Tokyo, Japan) according to a previous paper [38,41]. The radioactivity was determined with an auto well gamma counter and corrected for background radiation and physical decay during counting. A window from 16 to 69 keV was used for counting ^125^I, and a window from 70 to 120 keV was used for counting ^211^At. Correlation factors to eliminate any crossover of ^125^I activity in the ^211^At counts were determined by measuring the ^125^I standard in each window. More than one week after the experiments, the radioactivity counts of ^125^I were measured after the decay of ^211^At. The protein amount in cells was quantified using a BCA Protein Assay Kit (Nacalai Tesque, Kyoto, Japan) according to the manufacturer’s protocol. All data were expressed as percent dose per milligram protein (%dose/mg protein). In blocking experiments, a selective inhibitor of the PSMA, (2S)-2-(phosphonomethyl)pentanedioic acid (2-PMPA) (final concentration 100 µM), was added to each well with tracers. After incubation for 10, 60, and 120 min, radioactivity and protein concentration were determined in the same manner as the experiment without the blocking agent.

### 3.5. Biodistribution Experiments

Experiments with animals were conducted in strict accordance with the Guidelines for the Care and Use of Laboratory Animals of Kanazawa University. The experimental protocols were approved by the Committee on Animal Experimentation of Kanazawa University. The animals were housed with free access to food and water at 23 °C with a 12 h alternating light/dark schedule.

[^67^Ga]Ga-PSMA-617 (37 kBq) and [^125^I]**5** (37 kBq) or [^125^I]**5** (37 kBq) and [^211^At]**6** (37 kBq) were intravenously co-administered into three or four normal mice at each timepoint (6 weeks male ddY mice, 28–30 g, Japan SLC, Inc., Hamamatsu, Japan). Mice were sacrificed at 10 min, 1 h, 4 h, and 24 h ([^67^Ga]Ga-PSMA-617 and [^125^I]**5**) or at 10 min, 1 h, 4 h, 12 h, and 24 h ([^125^I]**5** and [^211^At]**6**) postinjection of the radiolabeled compounds. Tissues of interest were removed and weighed, and the radioactivity of ^67^Ga and ^125^I and ^125^I and ^211^At were determined with an auto well gamma counter (ARC-8001) and corrected for background radiation as described in the Cellular Uptake Experiments Section. To determine the amount and routes of the radioactivity excreted from the body for 24 h after the injection of radiolabeled compounds, mice were housed in metabolic cages (METABOLICA, SUGIYAMA-GEN Co., Ltd., Tokyo, Japan). Data are shown as the percentage of injected dose per gram of tissues (%ID/g) or %ID.

To evaluate the effects of an albumin-binding competitor (sodium salt of IPBA) in normal mice, [^125^I]**5** and [^211^At]**6** were intravenously co-administered. At 1 h postinjection of the radiolabeled compounds, IPBA (3.4 mg, 11 µmol) dissolved in saline at a 10 molar equivalent of blood albumin was administered [29]. Mice were sacrificed at 65 min, 70 min, 2 h, 4 h, 12 h, and 24 h postinjection of [^125^I]**5** and [^211^At]**6**. Tissues of interest were treated, and the radioactivity was measured in the same manner as above. Data are also shown as %ID/g or %ID.

### 3.6. Statistical Evaluation

Double-tracer experiments in the cellular uptake and animal experiments were compared using the paired Student’s *t* test. Blocking studies were compared using the unpaired Student’s *t* test. The level of statistical significance was set to *p*  <  0.05.

## 4. Conclusions

In this investigation, we evaluated the strategy of improving ABM-mediated pharmacokinetics, previously validated with RGD peptides, to determine if it is also applicable to PSMA ligands. By incorporating APBA into the PSMA-617 analog to produce [^211^At]**6**, we demonstrated that APBA effectively functioned as an ABM. Furthermore, its binding could be modulated through the administration of a competitor, indicating that this approach can be used to regulate the pharmacokinetics of ABM-containing compounds. These findings support the broader applicability of ABM-mediated pharmacokinetic modulations as a versatile strategy for radiotheranostics.

## Data Availability

The original contributions presented in the study are included in the article/Supplementary Materials; further inquiries can be directed to the corresponding author.

## Supplementary Materials

The following supporting information can be downloaded at: https://www.mdpi.com/article/10.3390/molecules30132804/s1, Scheme S1: Synthetic scheme of [^67^Ga]**5**; Figure S1: Time-dependent cellular uptake and internalization of [^125^I]**5** and [^67^Ga]**5** in LNCaP cells.; Table S1: Biodistribution of radioactivity after intravenous injection of [^125^I]**5** and [^67^Ga]Ga-PSMA-617 in normal mice.; Table S2: Biodistribution of radioactivity after intravenous injection of [^125^I]**5** and [^211^At]**6** in normal mice.; Table S3: Biodistribution of radioactivity 65, 70 min, 2, 4, 12, and 24 h after administration of [^125^I]**5** and [^211^At]**6** in normal mice. IPBA as a competitor was administered at 1 h postinjection of [^125^I]**5** and [^211^At]**6**.

## Abbreviations

The following abbreviations are used in this manuscript:

ABM	Albumin-Biding Moiety
APBA	4-(*p*-Astatophenyl)butyric Acid
^211^At	Astatine-211
BSA	Bovine Serum Albumin
DOTA	1,4,7,10-Tetraazacyclododecane-1,4,7,10-tetraacetic Acid
FBS	Fetal Bovine Serum
FDA	Food and Drug Administration
Fmoc	Fluorenylmethyloxycarbonyl
HPLC	High-Performance Liquid Chromatography
IPBA	4-(*p*-Iodophenyl)butyric Acid
ITLC-SG	Instant Thin-Layer Chromatography on Silica Gel
LNCaP	Lymph Node Carcinoma of the Prostate
mCRPC	Metastatic Castration-Resistant Prostate Cancer
MS	Mass Spectrometry
PBS	Phosphate-Buffered Saline
PET	Positron Emission Tomography
PSA	Prostate-Specific Antigen
PSMA	Prostate-Specific Membrane Antigen
RGD	Arg-Gly-Asp
RP-HPLC	Reversed-Phase High-Performance Liquid Chromatography
SPECT	Single-Photon Emission Computed Tomography
TAT	Targeted Alpha Therapy
TFA	Trifluoroacetic Acid
TIS	Triisopropylsilane
2-PMPA	(2S)-2-(Phosphonomethyl)pentanedioic Acid
