# Supplementary material for: Engineering a Radiohybrid PSMA Ligand with an Albumin-Binding Moiety and Pharmacokinetic Modulation via an Albumin-Binding Competitor for Radiotheranostics
Source: Molecules. 2025 Jun 29;30(13):2804. doi: 10.3390/molecules30132804 (PMC12251549; doi:10.3390/molecules30132804)
Supplement: Supplementary file 1 [file molecules-30-02804-s001.zip › molecules-3706796-supplementary.pdf]

Supporting Information

Engineering a Radiohybrid PSMA Ligand with an Albumin-Binding Moiety and Pharmacokinetic Modulation via an Albumin-Binding Competitor for Radiotheranostics

Saki Hirata [1], Hiroaki Echigo [1], Masayuki Munekane [1], Kenji Mishiro [1],
Kohshin Washiyama [2], Takeshi Fuchigami [1], Hiroshi Wakabayashi [3],
Kazuhiro Takahashi [2], Seigo Kinuya [3] and Kazuma Ogawa [1,*]

[1] Graduate School of Medical Sciences, Kanazawa University, Kakuma-machi, Kanazawa 920-1192, Ishikawa, Japan

[2] Advanced Clinical Research Center, Fukushima Global Medical Science Center, Fukushima Medical University, 1 Hikarigaoka, Fukushima 960-1295, Fukushima, Japan

[3] Department of Nuclear Medicine, Kanazawa University Hospital, Kanazawa University, Takara-machi 13-1, Kanazawa 920-8641, Ishikawa, Japan

***Corresponding Author**

Graduate School of Medical Sciences, Kanazawa University, Kakuma-machi, Kanazawa 920-1192; Japan

Telephone: 81-76-234-4460; Fax: 81-76-234-4460

E-mail: kogawa@p.kanazawa-u.ac.jp

Table of contents

Synthesis and radiolabeling of [^{67}Ga]**5**............ S3

In vitro stability S4

Cellular uptake of [^{125}I]**5** and [^{67}Ga]**5**............ S6

Biodistribution............ S7

Synthesis and radiolabeling of [^{67}Ga]5

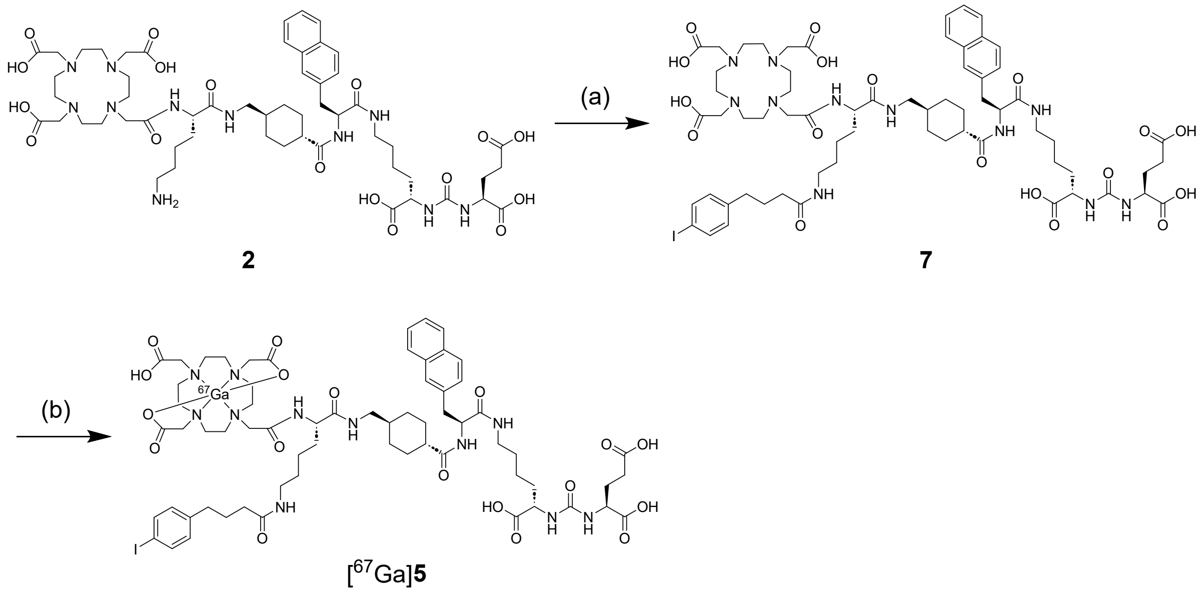

Scheme S1. Synthetic scheme of [^{67}Ga]**5**.

Reagents: (a) 4-(*p*-iodophenyl)butanoate NHS ester, DIPEA, DMF, 4 h, rt, (b) [^{67}Ga]GaCl_3, 3 M acetate buffer (pH 5.0), 15 min, 80 °C.

DOTA-K(IPBA)-TXA-Nal-KuE (7)

DOTA-K-TXA-Nal-KuE (**2**) (1.0 mg, 0.85 μmol, 1 equiv) was dissolved in 100 μL of DMF, and then 4-(iodophenyl)butanoate NHS ester (3.3 mg, 8.5 μmol, 10 equiv) and DIPEA (2.9 μL, 17.0 μmol, 20 equiv) were added to the solution. The mixture was stirred at room temperature for 4 h and purified by RP-HPLC performed with a Cosmosil 5C_{18}-AR-II (10 × 150 mm) at a flow rate of 4 mL/min with a gradient mobile phase of 60% methanol in water with 0.1% TFA to 80% methanol in water with 0.1% TFA for 20 min with UV detection at 254 nm. (Retention time: 9.4 min) The solvent

was removed by lyophilization to yield DOTA-K(IPBA)-TXA-Nal-KuE (**7**) (0.89 mg, 72%) as a colorless solid.

DOTA-K(IPBA)-TXA-Nal-KuE (**7**): HRMS (ESI$^+$) (m/z calcd for $C_{65}H_{93}IN_{11}O_{18}$ $[M+H]^+$): 1442.5739 found: 1442.5732.

[^{67}Ga]Ga-DOTA-K(IPBA)-TXA-Nal-KuE ([^{67}Ga]5)

Preparation of [^{67}Ga]**5** was performed according to previous reports with slight modifications [1, 2]. Compound **7** (30 μg) was dissolved in a mixed solution of 100 μL of 3 M acetate buffer (pH 5.0) and 10 μL of [^{67}Ga]$GaCl_3$ in 0.1 M aqueous HCl. After heating at 80 °C for 15 min, [^{67}Ga]**5** was purified by RP-HPLC using a Cosmosil 5C_{18}-AR-II (4.6 × 150 mm) at a flow rate of 1 mL/min with a gradient mobile phase of 60% methanol in water with 0.1% trifluoroacetic acid TFA to 80% methanol in water with 0.1% TFA for 20 min.

In vitro stability

To evaluate in vitro stabilities of [^{67}Ga]**5**, [^{125}I]**5,** and [^{211}At]**6** in 0.1 M phosphate buffer (PB) (pH 7.4), each tracer (111 kBq) was added to PB containing 10% ethanol. After the solutions were incubated at 37 °C for 24 h, the

samples were analyzed by RP-HPLC using a Cosmosil 5C_{18}-AR-II (4.6 × 150 mm) at a flow rate of 1 mL/min with a gradient mobile phase of 60% methanol in water with 0.1% trifluoroacetic acid (TFA) to 80% methanol in water with 0.1% TFA for 20 min. In vitro stability test in 0.1 M PB solution containing 10% ethanol was performed. The radiochemical purities of [^{67}Ga]**5**, [^{125}I]**5**, and [^{211}At]**6** were 93.1 ± 0.9%, 97.0 ± 0.7%, and 97.1 ± 0.9% (mean ± SD for three samples) after incubating at 37 °C for 24 h, respectively.

Cellular uptake of [^{125}I]**5** and [^{67}Ga]**5**

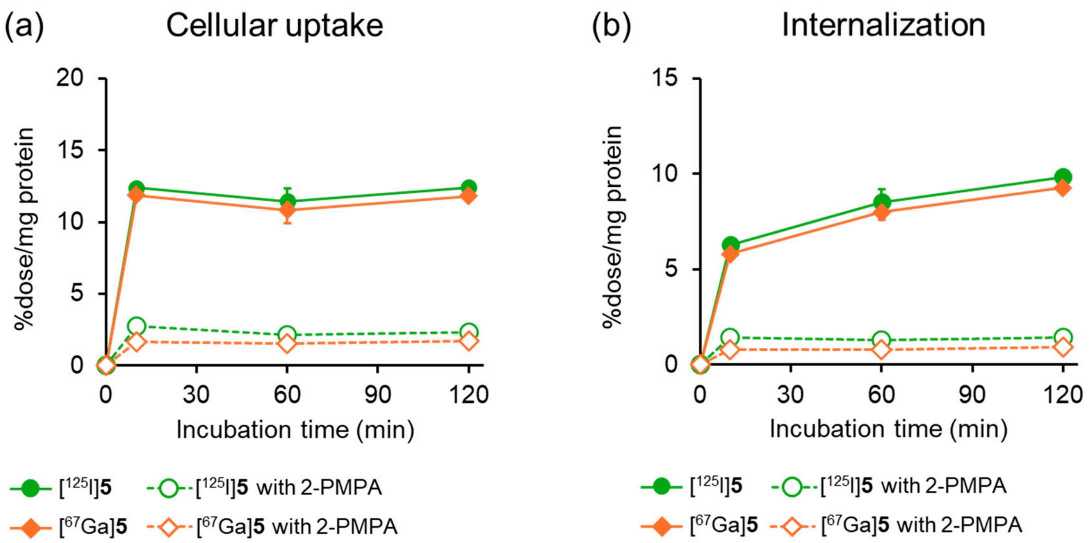

Figure S1. Time-dependent (a) cellular uptake and (b) internalization of [^{125}I]**5** and [^{67}Ga]**5** in LNCaP cells. Specific binding of PSMA is blocked by excess amount (100 μM) of 2-PMPA. Data are presented as mean ± SD for three samples.

Biodistribution

Table S1. Biodistribution of radioactivity after intravenous injection of [^{125}I]**5** and [^{67}Ga]Ga-PSMA-617 in normal mice.

Tissue	Time after injection			
	10 min	1 h	4 h	24 h
[^{125}I]**5**				
Blood	26.76 (0.56)	16.99 (1.80)	14.52 (0.22)	9.84 (0.85)
Liver	3.89 (0.34)	2.67 (0.11)	2.76 (0.11)	1.59 (0.34)
Kidney	10.29 (0.97)	6.22 (0.27)	6.93 (0.24)	7.37 (0.99)
S. Intestine	2.11 (0.10)	2.02 (0.21)	1.99 (0.14)	1.11 (0.31)
L. Intestine	1.10 (0.07)	1.16 (0.18)	2.03 (0.07)	1.15 (0.35)
Spleen	3.22 (0.38)	2.01 (0.29)	1.59 (0.35)	1.62 (0.16)
Pancreas	3.05 (0.20)	2.30 (0.22)	2.33 (0.13)	1.66 (0.46)
Lung	10.56 (0.97)	9.59 (2.25)	8.37 (0.62)	6.18 (0.83)
Heart	4.87 (0.63)	4.00 (0.35)	3.97 (0.22)	2.85 (0.36)
Stomach‡	0.78 (0.10)	0.75 (0.08)	0.69 (0.08)	0.67 (0.13)
Bone	2.56 (0.45)	2.18 (0.26)	1.57 (0.64)	1.57 (0.22)
Muscle	0.89 (0.02)	1.06 (0.06)	1.41 (0.07)	1.08 (0.11)
Brain	0.66 (0.14)	0.44 (0.19)	0.30 (0.03)	0.30 (0.03)
Neck‡	0.04 (0.01)	0.03 (0.01)	0.03 (0.00)	0.02 (0.00)
Urine‡				2.31 (0.33)
Feces‡				10.27 (0.73)
[^{67}Ga]Ga-PSMA-617				
Blood	3.40 (0.42)	0.29 (0.04)	0.07 (0.01)	0.01 (0.00)
Liver	0.74 (0.10)	0.18 (0.07)	0.08 (0.01)	0.04 (0.01)
Kidney	69.46 (8.76)	16.32 (3.17)	1.54 (0.08)	0.29 (0.06)
S. Intestine	0.75 (0.03)	0.16 (0.02)	0.22 (0.17)	0.04 (0.03)
L. Intestine	0.58 (0.07)	0.07 (0.01)	0.59 (0.79)	0.15 (0.11)
Spleen	0.96 (0.09)	0.14 (0.01)	0.05 (0.01)	0.03 (0.01)
Pancreas	1.03 (0.18)	0.14 (0.03)	0.04 (0.02)	0.02 (0.01)
Lung	2.60 (0.40)	0.28 (0.03)	0.06 (0.01)	0.02 (0.01)
Heart	1.22 (0.11)	0.11 (0.00)	0.04 (0.02)	0.01 (0.01)
Stomach‡	0.38 (0.02)	0.07 (0.02)	0.07 (0.04)	0.05 (0.04)
Bone	1.02 (0.23)	0.18 (0.04)	0.14 (0.05)	0.16 (0.04)

Muscle	0.75 (0.08)	0.09 (0.00)	0.02 (0.01)	0.01 (0.00)
Brain	0.11 (0.02)	0.02 (0.00)	0.01 (0.00)	0.00 (0.00)
Neck‡	0.02 (0.01)	0.00 (0.00)	0.00 (0.00)	0.00 (0.00)
Urine‡				2.40 (0.38)
Feces‡				58.01 (2.64)

Expressed as % injected dose per gram.

Each value represents the mean (SD) for three or four animals.

S. Intestine and L. Intestine mean small intestine and large intestine, respectively.

‡ Expressed as % injected dose.

Table S2. Biodistribution of radioactivity after intravenous injection of [^{125}I]**5** and [^{211}At]**6** in normal mice.

Tissues	Time after injection				
	10 min	1 h	4 h	12 h	24 h
[^{125}I]**5**					
Blood	24.34 (0.27)	15.32 (1.28)	12.08 (0.95)	11.77 (1.29)	8.71 (0.20)
Liver	3.56 (0.01)	2.32 (0.32)	1.50 (0.16)	1.59 (0.12)	1.29 (0.05)
Kidney	5.27 (0.62)	4.66 (0.50)	5.20 (0.25)	6.62 (0.34)	5.06 (0.61)
S. Intestine	1.63 (0.23)	1.62 (0.30)	1.28 (0.16)	1.08 (0.07)	0.69 (0.04)
L. Intestine	0.84 (0.07)	0.89 (0.15)	1.26 (0.30)	0.87 (0.13)	0.63 (0.09)
Spleen	2.78 (0.58)	1.79 (0.34)	1.54 (0.22)	1.75 (0.21)	1.21 (0.27)
Pancreas	2.73 (0.25)	1.86 (0.33)	1.57 (0.21)	1.75 (0.45)	1.35 (0.12)
Lung	13.97 (2.31)	8.89 (2.09)	7.01 (1.68)	8.00 (1.24)	5.00 (0.75)
Heart	4.48 (0.24)	4.10 (0.15)	3.88 (0.13)	3.62 (0.39)	2.91 (0.21)
Stomach‡	1.12 (0.10)	1.29 (0.50)	0.94 (0.29)	0.68 (0.09)	0.55 (0.02)
Bone	2.42 (0.54)	1.67 (0.13)	1.83 (0.61)	1.88 (0.25)	1.26 (0.12)
Muscle	1.01 (0.09)	1.24 (0.08)	1.20 (0.07)	1.32 (0.07)	1.08 (0.08)
Brain	0.50 (0.04)	0.33 (0.09)	0.29 (0.04)	0.34 (0.07)	0.22 (0.02)
Neck‡	0.06 (0.01)	0.15 (0.04)	0.27 (0.09)	0.12 (0.09)	0.17 (0.05)
Urine‡					2.51 (0.39)
Feces‡					8.13 (1.09)
[^{211}At]**6**					
Blood	26.93 (0.47)**	17.94 (1.58)**	13.40 (1.08)**	12.23 (1.51)	10.45 (0.19)***
Liver	3.61 (0.05)	2.54 (0.38)*	1.55 (0.24)	1.58 (0.12)	1.43 (0.07)**
Kidney	5.19 (0.64)	4.36 (0.53)*	4.44 (0.25)**	5.31 (0.31)***	3.50 (0.24)*
S. Intestine	1.42 (0.15)	1.59 (0.31)	1.30 (0.19)	1.06 (0.11)	0.81 (0.05)
L. Intestine	0.78 (0.05)*	0.93 (0.15)**	1.15 (0.35)	0.79 (0.16)*	0.62 (0.15)
Spleen	2.56 (0.44)	1.77 (0.11)	1.60 (0.25)	1.71 (0.33)	1.33 (0.43)
Pancreas	2.59 (0.24)	2.07 (0.45)	1.76 (0.35)	1.44 (0.82)	1.61 (0.22)*
Lung	15.25 (2.29)**	10.21 (2.55)*	7.81 (1.92)*	7.79 (0.46)	5.77 (0.84)**
Heart	4.66 (0.37)	4.88 (0.26)*	4.31 (0.39)	3.83 (0.57)	4.06 (1.41)

Stomach‡	0.85 (0.07)**	0.84 (0.27)	0.83 (0.18)	1.04 (0.11)*	1.31 (0.10)**
Bone	2.74 (0.53)	1.92 (0.21)	1.59 (0.09)	1.91 (0.32)	1.56 (0.33)
Muscle	0.99 (0.14)	1.25 (0.10)	1.27 (0.08)**	1.37 (0.10)	1.28 (0.09)*
Brain	0.53 (0.06)	0.38 (0.12)	0.32 (0.06)	0.42 (0.07)*	0.43 (0.14)
Neck‡	0.04 (0.01)	0.04 (0.01)	0.04 (0.01)*	0.18 (0.06)	0.14 (0.06)
Urine‡					1.31 (0.37)
Feces‡					2.00 (0.33)**

Expressed as % injected dose per gram.

Each value represents the mean (SD) for three or four animals.

S. Intestine and L. Intestine mean small intestine and large intestine, respectively.

‡ Expressed as % injected dose. *p < 0.05, **p < 0.01, ***p < 0.001 vs [^{125}I]**5**.

Table S3. Biodistribution of radioactivity 65, 70 min, 2, 4, 12, and 24 h after administration of [^{125}I]**5** and [^{211}At]**6** in normal mice. IPBA as a competitor was administered at 1 h postinjection of [^{125}I]**5** and [^{211}At]**6**.

Tissues	Time after injection					
	65 min	70 min	2 h	4 h	12 h	24 h
[^{125}I]**5**						
Blood	10.72 (0.60)	9.49 (0.85)	6.16 (0.73)	4.35 (0.53)	2.54 (1.16)	2.11 (1.41)
Liver	2.69 (0.27)	2.09 (0.38)	1.49 (0.17)	1.04 (0.12)	0.41 (0.15)	0.37 (0.23)
Kidney	19.55 (4.00)	29.15 (1.32)	40.70 (4.11)	30.73 (5.97)	14.90 (3.20)	7.40 (0.86)
S. Intestine	1.92 (0.17)	1.60 (0.19)	1.26 (0.11)	0.91 (0.19)	0.49 (0.12)	0.28 (0.21)
L. Intestine	1.19 (0.03)	1.19 (0.47)	1.07 (0.09)	1.19 (0.12)	1.11 (0.77)	0.47 (0.33)
Spleen	2.44 (0.29)	2.44 (0.62)	1.56 (0.42)	0.95 (0.11)	0.59 (0.18)	0.38 (0.17)
Pancreas	1.72 (0.29)	2.75 (1.08)	1.23 (0.07)	0.88 (0.18)	0.43 (0.17)	0.34 (0.19)
Lung	7.10 (0.83)	6.74 (0.50)	4.23 (0.42)	2.84 (0.36)	1.78 (0.66)	1.38 (0.82)
Heart	3.16 (0.26)	2.96 (0.25)	1.89 (0.27)	1.13 (0.13)	0.80 (0.31)	0.62 (0.41)
Stomach‡	1.25 (0.05)	1.19 (0.21)	0.88 (0.20)	0.51 (0.09)	0.71 (0.23)	0.21 (0.10)
Bone	2.03 (0.38)	2.21 (0.32)	1.08 (0.04)	0.82 (0.05)	0.71 (0.21)	0.41 (0.09)
Muscle	1.47 (0.08)	1.36 (0.15)	0.97 (0.12)	0.58 (0.02)	0.30 (0.09)	0.37 (0.32)
Brain	0.33 (0.06)	0.42 (0.09)	0.20 (0.05)	0.11 (0.07)	0.10 (0.02)	0.09 (0.04)
Neck‡	0.20 (0.03)	0.17 (0.03)	0.19 (0.02)	0.26 (0.11)	0.04 (0.03)	0.08 (0.07)
Urine‡						4.52 (3.20)
Feces‡						48.42 (7.50)

[^{211}At]**6**						
Blood	12.07 (0.88)*	10.98 (1.45)	7.50 (0.80)**	5.75 (0.86)	4.14 (1.25)**	3.65 (1.82)**
Liver	2.82 (0.23)	2.01 (0.42)	1.64 (0.22)*	1.28 (0.18)*	0.65 (0.16)***	0.58 (0.28)**
Kidney	16.94 (3.56)*	25.25 (0.96)**	32.27 (3.12)**	25.76 (3.95)	13.47 (3.38)	6.89 (1.53)
S. Intestine	1.58 (0.14)*	1.35 (0.14)*	1.32 (0.05)	1.11 (0.17)*	0.67 (0.17)*	0.47 (0.21)*
L. Intestine	1.29 (0.06)	1.03 (0.19)	0.93 (0.06)	0.98 (0.18)*	0.94 (0.47)	0.56 (0.33)**
Spleen	2.59 (0.28)*	2.45 (0.61)	1.42 (0.31)	1.08 (0.13)**	0.86 (0.22)	0.70 (0.15)*
Pancreas	1.73 (0.25)	1.68 (0.23)	1.32 (0.14)	1.17 (0.23)*	0.77 (0.32)*	0.41 (0.32)
Lung	7.86 (0.95)*	7.75 (0.58)**	5.22 (0.52)**	4.00 (0.51)**	3.19 (0.88)**	2.38 (1.01)**
Heart	3.49 (0.52)	3.39 (0.12)	2.15 (0.20)*	1.61 (0.15)**	1.36 (0.37)***	1.01 (0.48)**
Stomach‡	0.94 (0.07)	0.82 (0.11)**	0.77 (0.13)*	0.63 (0.13)**	0.59 (0.30)*	0.86 (0.21)**
Bone	2.29 (0.35)*	2.42 (0.39)	1.64 (0.07)**	1.00 (0.09)	0.69 (0.24)	0.38 (0.27)
Muscle	1.46 (0.08)	1.48 (0.28)	1.14 (0.15)*	0.76 (0.05)*	0.50 (0.21)	0.37 (0.20)
Brain	0.32 (0.05)	0.33 (0.12)	0.22 (0.04)	0.16 (0.05)	0.12 (0.04)	0.06 (0.03)
Neck‡	0.09 (0.04)*	0.06 (0.01)*	0.05 (0.01)*	0.07 (0.02)*	0.10 (0.08)	0.17 (0.06)*
Urine‡						3.49 (2.53)
Feces‡						28.38 (10.05)**

Expressed as % injected activity per gram. Each value represents the mean (SD) for three animals.

‡ Expressed as % injected activity. Significance was determined by paired Student's *t* test.

S. Intestine and L. Intestine mean small intestine and large intestine, respectively.

*$p < 0.05$, **$p < 0.01$, ***$p < 0.001$ vs [^{125}I]**5**.

References

[1] Echigo H, Munekane M, Fuchigami T, Washiyama K, Mishiro K, Wakabayashi H, et al. Optimizing the pharmacokinetics of an ^{211}At-labeled RGD peptide with an albumin-binding moiety via the administration of an albumin-binding inhibitor. Eur J Nucl Med Mol Imaging 2024;51:2663-71.
[2] Hirata S, Mishiro K, Higashi T, Fuchigami T, Munekane M, Arano Y, et al. Synthesis and evaluation of a multifunctional probe with a high affinity for prostate-specific membrane antigen (PSMA) and bone. Nucl Med Biol 2022;114-115:34-41.
